# Detection, Isolation and Confirmation of Crimean-Congo Hemorrhagic Fever Virus in Human, Ticks and Animals in Ahmadabad, India, 2010–2011

**DOI:** 10.1371/journal.pntd.0001653

**Published:** 2012-05-15

**Authors:** Devendra T. Mourya, Pragya D. Yadav, Anita M. Shete, Yogesh K. Gurav, Chandrashekhar G. Raut, Ramesh S. Jadi, Shailesh D. Pawar, Stuart T. Nichol, Akhilesh C. Mishra

**Affiliations:** 1 National Institute of Virology, Pashan, Pune, India; 2 Centers for Disease Control and Prevention, Atlanta, Georgia, United States of America; Tulane School of Public Health and Tropical Medicine, United States of America

## Abstract

**Background:**

In January 2011, human cases with hemorrhagic manifestations in the hospital staff were reported from a tertiary care hospital in Ahmadabad, India. This paper reports a detailed epidemiological investigation of nosocomial outbreak from the affected area of Ahmadabad, Gujarat, India.

**Principal Findings:**

Samples from 3 suspected cases, 83 contacts, Hyalomma ticks and livestock were screened for Crimean-Congo hemorrhagic fever (CCHF) virus by qRT-PCR of which samples of two medical professionals (case C and E) and the husband of the index case (case D) were positive for CCHFV. The sensitivity and specificity of indigenous developed IgM ELISA to screen CCHFV specific antibodies in human serum was 75.0% and 97.5% respectively as compared to commercial kit. About 17.0% domestic animals from Kolat, Ahmadabad were positive for IgG antibodies while only two cattle and a goat showed positivity by qRT-PCR. Surprisingly, 43.0% domestic animals (Buffalo, cattle, sheep and goat) showed IgG antibodies in the adjoining village Jivanpara but only one of the buffalo was positive for CCHFV. The *Hyalomma anatolicum anatolicum* ticks were positive in PCR and virus isolation. CCHFV was isolated from the blood sample of case C, E in *Vero E-6* cells and Swiss albino mice. In partial nucleocapsid gene phylogeny from CCHFV positive human samples of the years 2010 and 2011, livestock and ticks showed this virus was similar to Tajikistan (strain TAJ/H08966), which belongs in the Asian/middle east genetic lineage IV.

**Conclusions:**

The likely source of CCHFV was identified as virus infected Hyalomma ticks and livestock at the rural village residence of the primary case (case A). In addition, retrospective sample analysis revealed the existence of CCHFV in Gujarat and Rajasthan states before this outbreak. An indigenous developed IgM ELISA kit will be of great use for screening this virus in India.

## Author Summary

A nosocomial outbreak of CCHFV occurred in January 2011, in a tertiary care hospital in Ahmadabad, Gujarat State in western India. Out of a total five cases reported, contact transmission occurred to three treating medical professionals, all of whom succumbed to the disease. The only survivor was the husband of the index case. These results highlight the importance of considering CCHFV as a potential aetiology for Hemorrhagic fever (HF) cases in India. This also underlines the need for strict barrier nursing and patient isolation while managing these patients. During the investigation presence of CCHFV RNA in *Hyalomma anatolicum* ticks and livestock were detected in the village from where the primary case (case A) was reported. Further retrospective investigation confirmed two CCHF human cases in Rajkot village 20 kilometres to the west of Ahmadabad in 2010, and CCHFV presence in the livestock 200 kilometres to the north in the neighbouring State Rajasthan. This report shows the presence of CCHFV in human, ticks and animals in Gujarat, India. The fact of concern is the spread of this disease from one state to another due to trading of livestock.

## Introduction

Crimean-Congo hemorrhagic fever (CCHF) is a severe acute febrile illness caused by the CCHF virus (CCHFV, family *Bunyaviridae*, genus *Nairovirus*), with overall case fatality of 9–50% [1]. CCHF was first recognized in the Crimean peninsula in the mid-1940s, [2], but the virus was first isolated from a patient in Kisangani, Democratic Republic of Congo, in 1956 [3]. Person-to-person transmission of CCHFV occurs through direct exposure to blood or other secretions, and instances of nosocomial transmission are well-documented [1]. The virus is maintained in nature predominantly in the *Ixodid* tick vectors, particularly ticks of the genus *Hyalomma* [4], [5]. CCHFV can persist in the tick throughout its life stages by transtadial transmission, and can be passed onto the offspring by transovarial transmission [5]. Among domestic animals, cattle, sheep, and goat play an important role in the natural cycle of the virus [6]. In these animals, CCHFV replicates to high titres in the lung, liver, spleen, and reticuloendothelial system in other organs [7], but generally causes only subclinical disease. In contrast, human infections often result in severe hemorrhagic fever (HF), with high levels of viral replication occurring in all major organs, including the liver [8].

In recent years, a number of zoonotic viral diseases have emerged in Southeast Asia as Nipah virus, and CCHFV [9], [10], [11], [12]. CCHF was recently confirmed for the first time in India, although the virus had been identified nearby in Pakistan and western China [11], [12]. In January 2011, human cases with hemorrhagic manifestations in the hospital staff were reported from a tertiary care hospital in Ahmadabad, Gujarat. The clinical samples of three hospitalized patients were referred to National Institute of Virology (NIV), Pune and laboratory investigations confirmed as CCHFV [13]. Here, we report detection and isolation of CCHFV associated with that nosocomial outbreak in Gujarat, India, and the presence of the virus in livestock and ticks in this region. The disease is newly recognized in India. It is required to create awareness about this disease in public health workers and physicians. CCHFV symptoms are difficult to distinguish not only from other HF, but that the real challenge is to distinguish the signs and symptoms from other, more common, febrile diseases.

## Materials and Methods

### Ethics Statement

NIV, Pune is responsible for investigation of viral disease outbreaks of human including Zoonosis in India. As per the mandate of our institute, collection of the clinical samples from different species of animals for viral isolation and detection is required. This institute has policy to take approval on the projects which involves animals from the national committee called “Committee for the Purpose of Control & Supervision of Experiments on Animals (CPCSEA) under the Ministry of Environment and Forests, Government of India. Our study project No. HCL01/NIV15/2010 is approved by the Institutional Animal Ethical Committee (IAEC) permitting the use of infant and adult mice as laboratory animals for isolation of virus and development of antibodies respectively.

The present nosocomial CCHF outbreak was informed to Institutional Human Ethical Committee, NIV Pune. As per policy of institute the work conducted during epidemic situations is exempt from prior approval from the IEC. Due to this policy, this study was not pre-approved, but the Committee was notified after the outbreak. All study participants provided informed consent. Informed consent was in written format, both in English and local language (Guajarati). All the record analyzed was anonymized. Every sample was registered in the central registry of the institute and allotted a NIV number, which was used throughout the study.

#### Study area

Ahmadabad is a metropolitan city in Gujarat state, which shares international boundary with Pakistan. This communication reports confirmation of a focal nosocomial outbreak of CCHFV in a tertiary care hospital and finding of serosurvey in human and animals, RT-PCR and virus isolations from ticks, livestock and human.

### Identification of CCHF nosocomial outbreak and clinical sample collection

After the request from the Gujarat Government Public Health authorities, a team from the NIV, Pune visited the private tertiary care hospital in Ahmadabad, along with local public health authorities. Medical records of hospitalised patients presented with HF manifestations were examined, and their family members or caretakers were interviewed. Blood and urine samples were collected from cases with HF manifestations and their contacts. All clinical samples were transported in cold chain to NIV, Pune. To ascertain whether additional CCHFV infections had occurred in contacts (hospital staff or family members) lacking CCHF infection symptoms, an additional 86 blood samples including CCHF suspected cases were tested by Vector-Best and indigenously developed ELISA for detection of IgM antibodies against CCHFV.

### Identification of past CCHF cases in this region

Earlier, there were two serum samples referred to NIV, Pune from Rajkot, Gujarat, from patients suspected for Hantaan and Nipah virus in the month of February 2010. These samples were from the patient and consulting physician. Both had succumbed to the unknown infection. These samples were also included in the present study.

### Sample collection from rodents, domestic animals and ticks

Blood samples and ticks were collected from the buffalo, cattle, sheep and goat from the vicinity of the index case (case A), as well as from Kolat and surrounding villages, Jivanpura, Navapura and Changodar. A total of 138 ticks were collected from domestic animals from Kolat, were classified and pooled as per species. Eight tick pools were made and homogenized in MEM media. Tick homogenates were suspended in lysis buffer for viral RNA extraction. These were tested for CCHFV by qRT-PCR and nested RT-PCR. Hyalomma tick homogenates were also used for virus isolation in *Vero E-6* cells and Swiss albino mice.

Rodents (n = 90) were also trapped from Kolat villege, morphologically identified and only blood samples from these animals were collected and transported to NIV, Pune.

### Detection of CCHFV by qRT-PCR, nested RT-PCR and virus isolation

RNA was extracted from human (serum and urine), and animal serum samples using Qiagen (Valencia, CA, USA) RNA extraction kit. Tick pools were homogenized in Minutesimum Essential Medium (MEM). This homogenate was used for RNA extraction and for virus isolation. In the initial screening CCHFV-specific TaqMan based qRT-PCR was carried out on the RNA as previously described [14]. RT-PCR was performed with the SuperScript One-Step RT-PCR kit with Platinum Taq (Invitrogen Corp., Carlsbad, CA, USA). Two sets of primers were used for initial RT-PCR. The primer set CCHF-F2 (TGG ACA CCT TCA CAA ACT C) and CCHF-R3 (GAC AAA TTC CCT GCA CCA) amplified a 530 nt region of the nucleocapsid (N) gene of CCHFV, while nested PCR using the primers CCHF-F3 (GAA TGT GCA TGG GTT AGC TC) and CCHF-R2 (GAC ATC ACA ATT TCA CCA GG) amplified a 226 nt region [15]. PCR products were analyzed on 2% agarose gel electrophoresis and Ethidium bromide straining. Cyclic sequencing was carried out at PCR condition 96°C - 1 minute, 96°C - 10 sec, 45°C - 5 sec and 60°C - 4 minutes for 25 cycles using ABI Big-Dye 3.1 dye chemistry (Applied Biosystems, Foster City, CA). These products were purified using Dyex 2.0 kit (Qiagen) according manufacturer's instructions and sequencing was performed using the ABI 3100 automated DNA sequencer. The sequences obtained were curated using KODON software for both the reads from both the ends. The curated sequences were aligned using program Clustal W and phylogenetic tree was constructed using neighbour joining algorithm with 500-bootstrap replicates as implemented in Mega v 4.0 software [16].

The tick pools were first tested by qRT-PCR. Homogenates of CCHFV-positive tick pools were inoculated into *Swiss albino* mice via intracerebral and intraperitoneal routes and into *Vero E6* cells for virus isolation. Virus isolation was attempted from the CCHF positive human blood, serum, and urine samples.

### IgM capture ELISA for screening of human samples

Two CCHF IgM ELISA kits were used; a) commercial kit, b) indigenously developed test for detection of IgM antibodies in the human serum samples.

#### a. Commercial kit

A commercial kit (Vector BEST Company, Vectocrimean-CHF IgM kit, Novosibirsk, Russia) was used and the protocol followed as per the manufacturer's instructions.

#### b. Indigenously developed test for CCHF IgM detection

An IgM capture ELISA was developed for serological diagnosis of CCHFV infection from patient's serum. Briefly, ELISA wells were coated with anti-human IgM antibodies (dilution 1∶100) (Invitrogen AHI0601) in carbonate buffer (pH 9.2, 0.025 Molar) overnight at 4°C. These wells were blocked with 2% skimmed milk powder in 10 mM PBS pH 6.8. Coated and blocked wells were added with 100 ul of 1∶100 diluted serum samples and incubated at 37°C for one hr. β-Propiolactone (BPL) inactivated CCHFV infected *Vero* cell lysate antigen (1∶20 diluted, 100 µl/well) was added as a positive antigen, normal *Vero* cell lysate was used as negative antigen and incubated for one hr at 37°C. These wells were washed and anti CCHFV antibody raised in mice (1∶4000 diluted, 100 µl/well) was added further incubated one hr at 37°C. For Anti mouse HRP conjugate (1∶4000 diluted, 100 µl) (Pierce Cat No 31446) was added and incubated for one hr at 37°C. ABTS substrate was added and incubated for 25 minutes. The reaction was stopped by adding 1% SDS and plates were read at 414 nm. The plates were washed five times using 10 mM PBS pH 7.4 with 0.1% Tween-20 (Sigma, USA) at the end of each step. Appropriate controls were included in the test. CCHFV inactivation treatment was done in BSL 3 lab, and following inactivation, the sample was handled in BSL 2 laboratory for ELISA testing.

### IgG capture ELISA for screening of animal samples

The inactivated animal serum samples were tested for evidence of anti-CCHFV IgG using an ELISA kit provided by CDC, Atlanta. The protocol followed was, ELISA plates were coated with anti-CCHFV hyper immune mouse ascetic fluid (HMAF) (dilution 1∶1000) in phosphate buffer saline pH 7.4 overnight at 4°C. BPL inactivated CCHFV infected *Vero E6* cell lysate antigen (1∶20 diluted, 100 µl/well) was added as a positive antigen, normal *Vero E6* cell lysate was used as negative antigen and incubated for one hr at 37°C. One hundred µl of 1∶100 diluted serum samples were added and incubated for one hr at 37°C. These wells were washed and anti-sheep IgG HRP conjugate (1∶4000 diluted, 100 µl) was added and incubated for one hr at 37°C. ABTS substrate was added and incubated at 37°C for 30 minutes. The reaction was stopped by adding 1% SDS and plates were read at 414 nm. The plates were washed five times using 10 mM PBS pH 7.4 with 0.1% Tween-20 (Sigma, USA) at the end of each step. Appropriate controls were included in the test. During investigation, ELISA was also performed to detect CCHFV specific IgG antibodies in the animals from the residential area and surrounding villages of index case (case A).

## Results

### Identification of CCHF nosocomial outbreak

In December 2010 and January 2011, a cluster of viral hemorrhagic fever (VHF) cases was identified in Ahmadabad, Gujarat, India, which was declared as a nosocomial outbreak of Crimean-Congo hemorrhagic fever [13]. The initial case identified was a 25 year old nurse (case C) who worked in a hospital in Ahmadabad, and presented on January 13th, 2011 with a three days history of an acute febrile illness characterized by fever, chills, vomiting and headache followed by hemorrhagic symptoms [13]. Her condition rapidly deteriorated with onset of delirium, multiple hemorrhagic symptoms (palatal petechia, coughing up of blood, bleeding from lips, vaginal bleeding, hematuria, hematemesis, and pulmonary hemorrhage). She was placed in isolation on January 16th and given oral ribavirin based on suspicion of VHF. Despite treatment, she died on January 18th from multi-organ failure and disseminated intravascular coagulation (Table 1). Based on the suspicion of VHF, CCHFV was added to the diagnostic testing and the patient sera tested positive by qRT-PCR and RT-PCR in urine and serum [13].

**Table 1:** Case descriptions and characteristics of CCHF cases treated in tertiary care hospital at Ahmadabad.

Variable	probable case A	probable case B	case C	case D*	case E
Occupation	Housewife	Physician	Nurse	Farmer	Physician
Age [Years]	32	35	25	32	25
Gender	Female	Male	Female	Male	Male
Date of onset of illness	December 27, 2010	January 6. 2011	January 10. 2011	January 14. 2011	January 26. 2011
Case descriptions	1 POD: Moderate fever, joint pain, headache, tachycardia, and myalgia received symptomatic treatment.3 POD: Severe vomiting, epigastric pain, distended abdomen, and admitted to a tertiary care hospital.5 POD: Altered sensorium and ascitis and admitted to a medical intensive care unit, treated conservatively with IV fluids, antacids, antibiotics, antiemetics, and other supportive treatment.7 POD: Oliguria, anaemic, breathlessness, acute liver failure. Died, cardio respiratory arrest and multi-organ failure.	1 POD: High grade fever and watery diarrhoea2 POD: vomiting, severe headaches, and delirium4 POD: high grade fever with delirium and altered sensorium, admitted to a private hospital.5 POD: Eye suffusion and hematoma on right inguinal region and thigh6 POD: Condition worsened, referred to another private hospital7 POD: Suddenly went into cardiac arrest and died	1 POD: High-grade fever with chills, vomiting, and headache3 POD: Haemoptysis, bleeding from the lips, hematuria, palatal petechiae, hematemesis6 POD: breathlessness7 POD: Drowsy and disoriented and mild ascitis, melena, vaginal bleeding, and plural effusion, pulmonary hemorrhage. Moderate hepatomegaly with diffuse hypoechoic parenchymal echotexture, and a contracted gall bladder with diffuse edematous wall.8 POD: Died from multi-organ failure and disseminated intravascular coagulation.	1 POD: High fever with chills, vomiting2 POD: joint pain, epigastric pain with tenderness and severe weakness3 POD: Admitted to same tertiary care hospital, given Ribavirin and other supportive medication10 POD: recovered and discharged on January 27, 2011.	1 POD: High grade fever with rigors, severe headache, vomiting2 POD: Severe weakness4 POD: Mild abdominal pain, signs of dehydration, tachycardia5 POD: Abdominal distension and tenderness, sevear haemetemesis, splenomegaly.6 POD: Gestrointestinal bleeding and tachypnoea, resulted death.
Incubation period [Probable]	Uncertain	7	9	12	Uncertain
Most likely exposure	Ticks or livestock at residence	Percutaneous/direct contact from body secretion of probable Case A	Percutaneous/direct contact from body secretion of probable Case A	Percutaneous/direct contact from body secretion of probable Case A	Case B & D contact; timing of disease onset suggests Case D as likely exposure source
Ribavirin therapy within 4 days of onset of symptoms	No	No	No	Yes	Yes
Diagnosis	Probable CCHF as husband [Case D] confirmed CCHF	Probable CCHF as contact with probable case A	Laboratory confirmed CCHF	Laboratory confirmed CCHF	Laboratory confirmed CCHF
Outcome & date	Death [3-Jan-11]	Death [13-Jan-11]	Death [18-Jan-11]	Recovered [27-Jan-11]	Death [31-Jan-11]

*:  = Husband of index case.

**NB:** Clinical laboratory data are mentioned in the Table 2.

Epidemiologic investigation revealed that case C had earlier provided care to a patient (case A) with similar VHF symptoms. Probable case A was a 32 year old housewife from Kolat village, approximately 20 km outside Ahmadabad. She had been admitted to the hospital on December 27^th^, 2010 and had died on December 31st with VHF-like symptoms (Table 1). While no specimens remained from case A to allow confirmatory testing for the presence of evidence of CCHF infection, case A was strongly suspected to be the source of nosocomial infection of the attending nurse (case C) given the similarity in clinical and laboratory findings (Table 2). Further investigation revealed a similar VHF-like illness in a 42 year old physician (probable case B), who had treated probable case A and had subsequently presented with symptoms on January 6th 2011 (Table 1) and died on January 13th. Virus transmission to two further contacts was documented. The husband (case D) of probable probable case A was admitted to the hospital on January 16th, displayed similar VHF-like symptoms, was treated with oral ribavirin, and recovered and was released on January 26th. Laboratory testing confirmed evidence of CCHF virus infection. The last identified case was a 25 year old doctor (case E) who had contact in the hospital with probable case B and case D. Given his onset of illness on January 26th, case E was likely exposed to case D, who had been admitted on January 16th and released on the 26th (and case B had died on January 13th). Despite oral ribavirin treatment, case E progressed to multi-organ failure and died on January 31st [13].

**Table 2:** Laboratory data on biochemical and microbiological parameters in suspected CCHF cases during admission to hospital.

Test	Unit/Reference	Case A (Index case)	Case B	Case C	Case D	Case E
WBCs	4000–10,000/CMM	3500	5310	3120	3000	5860
Platelet Count	150000–500000/CMM	5000	59100	50200	104000	27900
Lymphocytes	20–45%	17	11	22	10	12
Ferritin serum	10–291 ng/ml	ND	143729	40000	3866	ND
Alanine transaminase [SGPT]	9–52 U/L	2186	453	734	258	165
Blood Urea	14–36 mg/dl	63	37	ND	32	85
Serum LDH	313–618 U/L	23349	1	491	1921	3746
Serum Creatine phosphokinase [Total]	38–174 U/L	45	928	297	333	1027
Aspartate transaminase [SGOT]	14–36 U/L	ND	334	ND	195	463
Serum Bilirubin (Total)	0.2–1.3 mg/dl	1.5	0.75	1.98	3.29	2.5
Serum Bilirubin (Direct)	0–0.3 mg/dl	1.3	0.1	1.26	1.55	0.36
Serum Bilirubin (Indirect)	0–1.1 mg/dl	ND	0.6	0.72	1.23	1.8
Serum Alkaline Phosphatase	38–120	ND	257	ND	173	ND
Fibrinogen	180–350 mg/dl	80	80	376	ND	1.6
Prothrombin Time Test	11–16 Sec	34	28	25	25	20

*: ND : Not Done.

In total, 3/5 of these hospitalized individuals were confirmed by qRT-PCR, nested RT-PCR & IgM ELISA as having been infected with CCHFV. The patients initially reported fever, headache, myalgia and vomiting. Death occurred within 5–9 days POD in 4 of the 5 patients. Analysis showed that all the probable cases presented with similar laboratory data as laboratory confirmed CCHF cases, including leukopenia, thrombocytopenia, increased SGOT and SGPT and serum LDH, and increased ferritin and prothrombin time (Table 2). None of blood samples were found positive by qRT-PCR from the family and hospital contacts.

### Identification of past CCHF cases in this region

With the finding of CCHFV associated with acute hemorrhagic diseases in Gujarat, retrospective analysis was carried out on some samples collected in February, 2010 from patients that showed similar case descriptions and laboratory findings as reported here for these Ahmadabad cases. Acute blood samples from two patients from Rajkot, a town approximately 200 km west of Ahmadabad, were found positive for CCHFV by qRT-PCR and RT-PCR, retrospectively confirming them to be CCHF cases.

### Detection of CCHFV specific IgM antibodies in human samples

Out of 86 samples one hospital and one family contact (both asymptomatic) was positive for presence of IgM antibodies against CCHFV. Case C and E had a very high titter of IgM in the serum samples while case D was negative in the early sample which was positive for qRT-PCR. The 2nd sample of case D was positive for CCHFV specific IgM and also qRT-PCR. The indigenously developed ELISA was compared with commercial Vector-Best assay. The sensitivity and specificity of indigenous ELISA was 75.0% and 97.5% respectively using Vector-Best assay as gold standard (Table 3). The serum samples of case C, D, and E were found positive for IgM antibodies while screening with both the kits. As positive samples were only four, further standardization of indigenously developed assay using more human serum samples is required.

**Table 3:** Comparison of indigenously developed and commercial IgM capture ELISA for detection of antibodies.

Result by NIV kit	Result by Commercial kit		Total
	Positive	Negative	
Positive	3	2	5
Negative	1	80	81
Total	4	82	86

**Note:** 2×2 table for comparison of sensitivity and specificity of NIV and Vector-Best ELISA tests (Sensitivity = 3/4 = 75.0%; Specificity = 80/82 = 97.5%).

### Virus isolation from CCHFV positive human samples

The blood and urine samples collected from the laboratory confirmed CCHF cases (case C, D and E) were inoculated into *Vero E6* cells and *Swiss albino* mice [intra cerebral. and intra peritonial. routes]. Sickness and death was observed in mice on 9^th^ post infection day (PID). Cytopathic effect was observed in *Vero E 6* cells at 3^rd^ PID and virus was harvested on 7^th^ PID. CCHFV was isolated from the blood samples in both mice and *Vero E6* cells and sequence analysis confirmed that the PCR products were derived from CCHFV RNA. No virus isolate was obtained from the urine samples; the Ct values (34) of the qRT-PCR also suggested low viral titres in the urine.

### CCHFV circulation and detection of IgG antibodies in livestock

**Figure 1:**
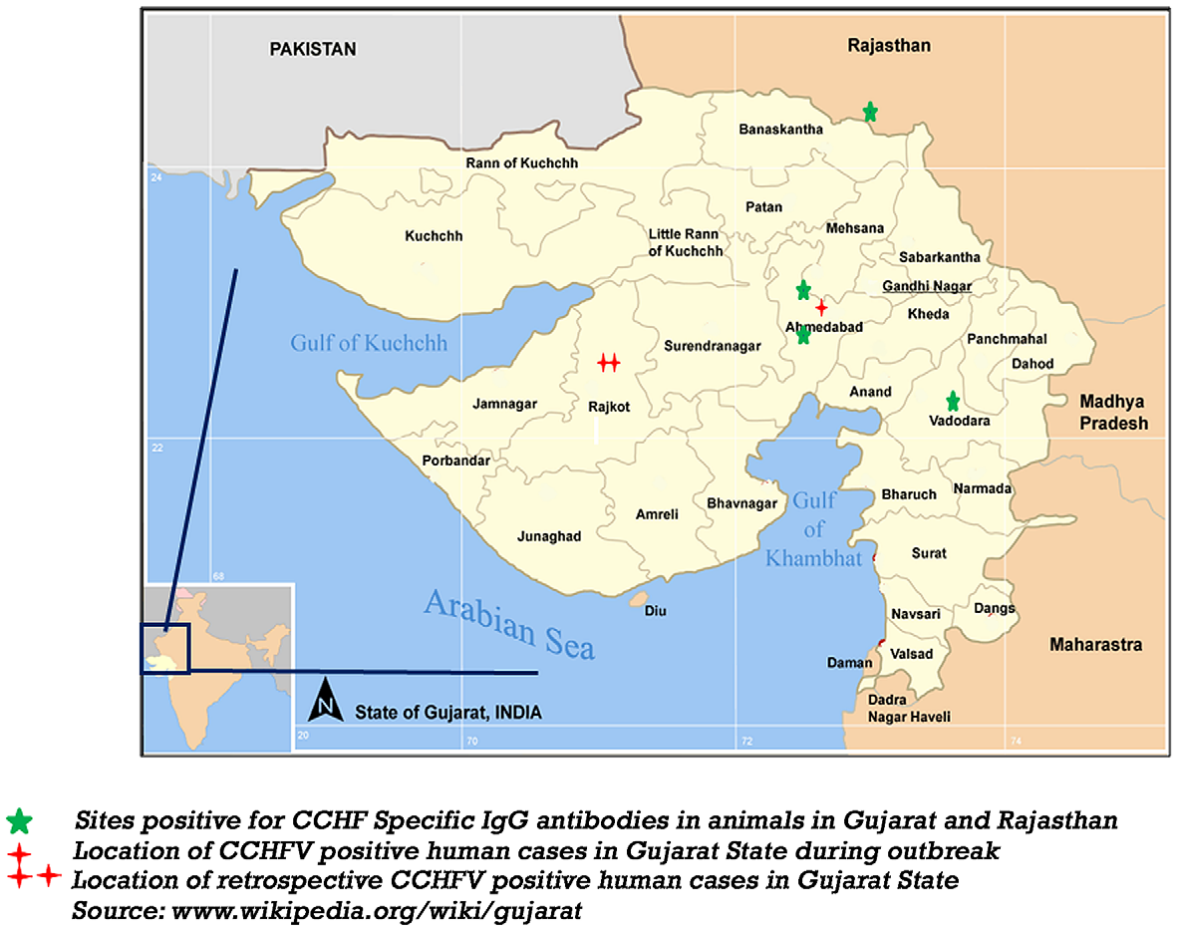
CCHF positive human cases and IgG antibody positive animals from Gujarat and Rajasthan state.

The initial virus source for this person to person chain of transmission appeared to be probable case A. She and her husband (case D) were reside in the rural village of Kolat, made a living by farming and have daily exposure to livestock and potentially ticks on these animals (Figure 1). In November and December 2010, just prior to this outbreak, a serosurvey was performed to examine livestock for evidence of CCHFV using blood samples collected from slaughterhouses in the northern adjoining state of Rajasthan and some more distant areas from Maharashtra and West Bengal states. The serum samples from buffalo, goat and sheep from Sirohi district, in southern Rajasthan were positive for IgG antibodies against CCHFV. Serum samples from northern West Bengal or Pune area of Maharashtra were negative (Table 4). The area from which positivity was reported was approximately 200 kilometres north of Ahmadabad (Figure 1).

**Table 4:** Cross-sectional survey of anti-CCHFV IgG in animals from three Indian states (November–December, 2010).

Animal	Number tested	Number positive for CCHFV IgG
Pune area, Maharashtra		
Buffalo	31	00
Goat	68	00
Pig	25	00
Sheep	08	00
Total (a)	132	00
North West Bengal		
Cattle	05	00
Goat	68	00
Total (b)	73	00
Sirohi district, Rajasthan		
Buffalo	15	01
Goat	10	03
Sheep	09	06
Total (c)	34	10 (29.4%)
Total (a+b+c)	239	10 (4.1%)

Evidence of CCHFV infection (IgG positive) was also found in a follow-up study of livestock [including buffalo, cattle, goat, and sheep] from Kolat (residence of case “A” and case “D”) and the surrounding villages of Changodar, Jivanpara and Navapura (Table 5). Overall IgG antibodies positivity in the small sample sizes varied between villages, (10–43%) (Table 5). qRT-PCR analysis showed evidence of CCHFV infection in the blood samples from three animals [two cattle and a goat] indicating circulation of the virus in these animal populations (data not shown) which was further confirmed by CCHFV specific nested RT-PCR.

**Table 5:** Anti-CCHFV IgG antibody positivity in animals from four villages in Ahmadabad.

Village	Animal	Total tested	IgG positive	Percent positivity
Changodar	Buffalo	15	2	
	Cattle	8	1	
	Goat	36	3	
	Sheep	1	0	
	Total	60	6	10.00
Jivanpara	Buffalo	9	2	
	Cattle	10	1	
	Goat	20	11	
	Sheep	30	16	
	Total	69	30	43.48
Kolat	Buffalo	75	16	
	Cattle	52	1	
	Goat	20	9	
	Sheep	1	0	
	Total	148	26	17.57
Navapura	Buffalo	24	4	
	Cattle	4	0	
	Total	28	4	14.29
Grand Total	305	66	21.6	

Rodent samples from *Rattus rattus rufescens* (72), *Suncus murinus* (6), *Mus booduga* (3), *Mus musculus* (8) and *Rattus norvegicus* (1) were tested by qRT-PCR for detection of CCHFV. All the 90 blood samples were negative for the presence CCHFV.

### CCHF virus circulation and detection in *Hyalomma* ticks

Out of eight tick pools collected from the livestock around the Kolat village two pools, both *Hyalomma anatolicum* were positive by qRT-PCR and RT-PCR specific to CCHFV (Table 6). These two Hyalomma tick pools also showed CPE in *Vero E-6* cells and propagated in *Swiss albino* mice also.

**Table 6:** CCHFV detection in ticks from Kolat village, Sanand Taluka, Ahmadabad, Gujarat.

Pool #	qRT-PCR	Tick species [host]	Tick sex [n]
1	Negative	*Hyalomma anatolicum* [Buffalo]	Female [19]
2	**Positive**	*H. anatolicum anatolicum* [Buffalo]	Male [31]
3	Negative	*H. anatolicum anatolicum* [Buffalo]	Nymph [10], Females [5]
4	Negative	*H. anatolicum anatolicum* [Buffalo]	Male [20], Female [4]
5	**Positive**	*H. anatolicum anatolicum* [Buffalo]	Male [13], Female [1]
6	Negative	*Ornithodoros savygni* [Cattle shed soil]	Nymph [23]
7	Negative	*O. savygni* [Cattle shed soil]	Male [10]
8	Negative	*O. savygni* [Cattle shed soil]	Female [2]
Grand Total			138

### Phylogenetic analysis

**Figure 2:**
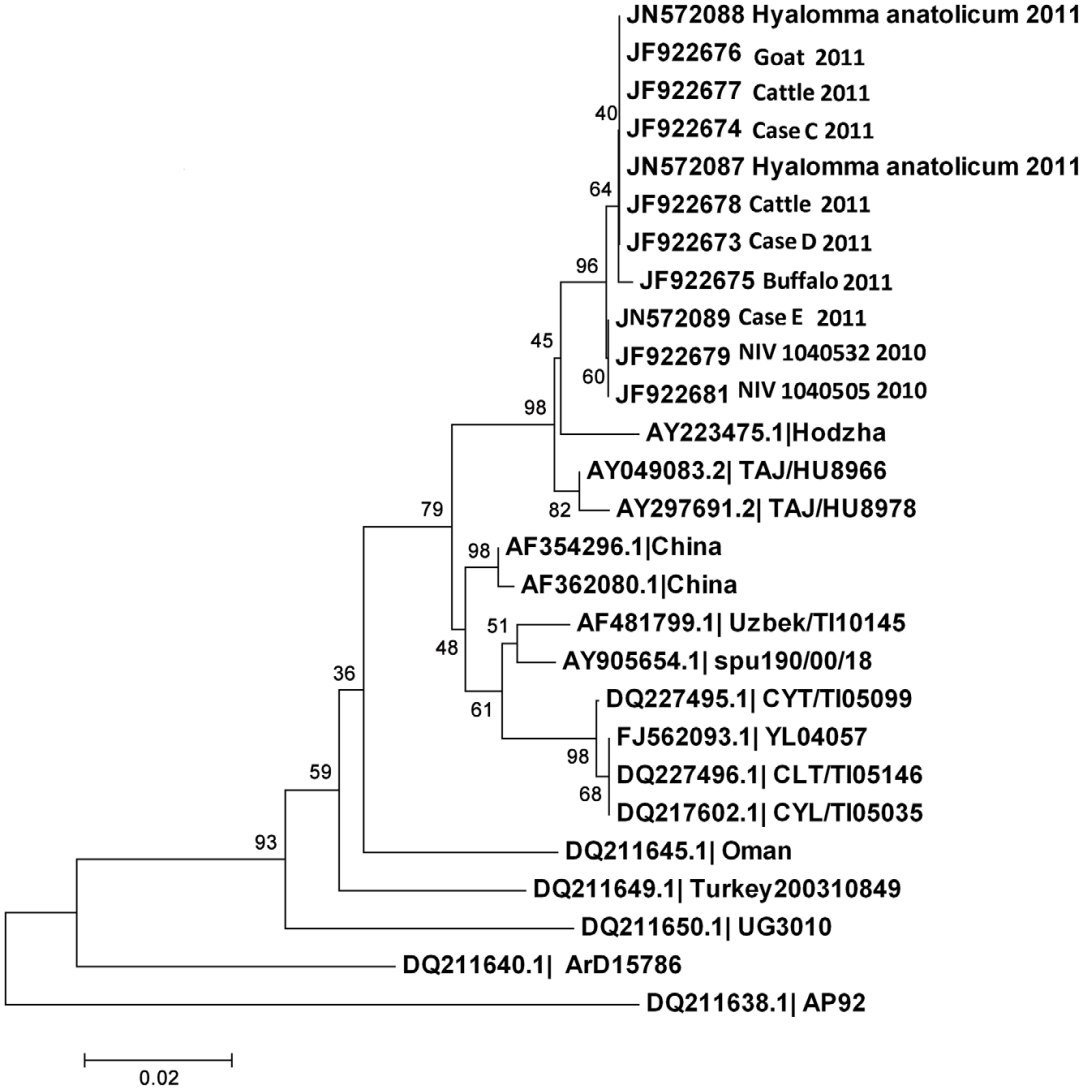
Phylogeny tree of CCHFV sequences from India based on partial nucleocapsid gene.

CCHFV specific nested RT-PCR products were generated from the blood samples from CCHF cases C, D and E of this outbreak and two retrospective serum samples of year 2010. Along with this, sequences of CCHFV positive two tick pools, and four livestock samples collected in Kolat village were also included for the analysis. Sequencing analysis of the PCR products generated a 226 nt partial fragment of the virus S gene. Comparison of this virus sequence fragment with that of other CCHFVs showed that the strain detected in Ahmadabad had maximum nucleotide identity (98.0%) with Tajikistan strain (TAJ/H08966) of CCHFV which belongs in the Asian/middle east genetic lineage IV (Figure 2).

## Discussion

India did not report any CCHF cases until January 2011 [13]. However, the presence of CCHFV in India had been suspected, since it was detected in the neighbouring countries of Pakistan and western China, especially once CCHFV was first isolated from the tick species *Hyalomma anatolicum* and from a mixture of *Hyalomma* and *Boophilus* tick species collected in Pakistan [17], [18]. In the past a serosurvey was conducted in Jammu and Kashmir, the western border districts of India (1976), showed CCHFV antibodies in many of the animal sera, however, studies by Rodrigues et. al., (1986) precipitated the antibody in 34 of the 655 domestic animal sera from different states/territories of southern India, and from Maharashtra state. Most of the positive sera were collected from goat in southern India [19].

CCHFV IgG antibodies were detected in animals in November and December 2010, in the neighbouring Rajasthan state in buffalo, cattle, goat, and sheep. The border between India and Pakistan is porous for the entry of livestock animals, which might play an important role in transmission of CCHFV between these countries. Our investigation identified CCHFV in cattle, goat, and buffalo from surrounding villages also. These villages have a large buffalo population. Since this virus is isolated from the ticks collected from buffalo in the affected area, these animals may have an important role in infecting large number of ticks in these areas. The pool of male *Hyalomma anatolicum* ticks was positive for CCHFV, suggesting that this disease is not due to recent introduction of CCHFV to this area. The virus strain studied here showed highest homology (98–100.0%) with the Tajikistan strain (TAJ/H08966), suggesting Asian/middle east origin (IV clade).

Among the CCHF confirmed cases, serum ferritin levels were high; in the fatal cases, such high ferritin levels have also been reported as indicator of severity of the disease [20]. Leukopenia, thrombocytopenia, increased SGOT, SGPT, LDH, and increased prothrombin time was in accordance to the World Health Organization (WHO) case definition of CCHF. The increased serum creatine phosphokinase is perhaps indicative of severe damage to the liver and other organs. Swiss albino mice inoculated with patient serum also showed intra-abdominal bleeding, multifocal lung necrosis, liver enlargement, and necrosis. Interestingly, blood smear examination reports from all patients showed erythophagocytosis, and investigations of mice inoculated with patient blood samples revealed similar findings. Case-D, who recovered 10 post onset days (POD) and was monitored for the presence of virus in the urine, and found positive for CCHF up to 13 days POD. This raises the question whether a recovered patient discharged before 13–14 days POD can still be a potential source of infection.

The short incubation period and many of the nonspecific symptoms of CCHF, which overlap with the symptoms of other hemorrhagic fevers, raise the risk for misdiagnosis and person-to-person transmission of CCHFV. This puts close contacts, and healthcare providers at risk for secondary infection. The symptoms, signs, and laboratory abnormalities of CCHF are nonspecific and can overlap with those of other tropical infections (dengue, Kyasanur Forest disease and leptospirosis). The efficacy of ribavirin is still debated, and it is difficult to say whether the only surviving CCHF case (husband of index case) in the present study was due to immediate ribavirin oral therapy. These features highlight the importance of quick diagnosis of the etiologic agent associated with HF cases. Moreover, once CCHF is suspected, patients and specimens should be handled with adequate biosafety measures. Authorities and contacts should be immediately notified, and patients' samples should be sent for specific laboratory diagnosis, keeping in view the precautions associated with BSL 4 risk group agent hazards.

Nosocomial transmission in this outbreak as reported here and earlier by Mishra et. al., (2011) is evident by the development of disease in close contacts of a CCHF patient [13]. This episode of nosocomial infection resulted in four deaths in a small population as of February 15, 2011. There are very few commercially available kits for detection of CCHFV antibodies from humans. Therefore, an attempt was made to develop indigenous assays for detection of CCHFV antibodies. The performance of indigenous assay was satisfactory as compared to commercially available Vector-Best assay. Since, the positive samples were only four, further standardization of indigenously developed assay using more human serum samples is required. Detection of IgM antibodies in one each hospital and family contacts suggest a low level of asymptomatic cases. The CCHF positivity of the two retrospective human samples from Rajkot, Gujarat also indicate that disease was prevalent in this state for a long time and probably might have contributed significant morbidity and mortality in the past in this state.

## Supporting Information

Checklist S1 STOBE checklist.
(DOC)
